# Observation of Excitonic Doublet Structure, Biexcitons and Their Temperature Dependence in High-Quality β-InSe Single Crystals

**DOI:** 10.3390/ma18194451

**Published:** 2025-09-23

**Authors:** Tran Thi Thu Huong, Long V. Le, Nguyen Thu Loan, Man Hoai Nam, Tien-Thanh Nguyen, Thi Thuong Huyen Tran, Ung Thi Dieu Thuy, Thi Huong Nguyen, Tae Jung Kim

**Affiliations:** 1 Institute of Materials Science, Vietnam Academy of Science and Technology, Hanoi 100000, Vietnam; huongttt@ims.vast.ac.vn (T.T.T.H.); loannt@ims.vast.ac.vn (N.T.L.); nammh@ims.vast.ac.vn (M.H.N.); ntthanh@ims.vast.ac.vn (T.-T.N.); huyenttt@ims.vast.ac.vn (T.T.H.T.); dieuthuy@ims.vast.vn (U.T.D.T.); 2 Department of Physics, Nha Trang University, Nha Trang 650000, Vietnam; 3 Department of Physics, Kyung Hee University, Seoul 02447, Republic of Korea

**Keywords:** InSe single crystal, Mott-Wannier excitons, biexcitons

## Abstract

We present a systematic study of the fundamental optical properties of indium selenide (InSe) single crystals over a temperature range of 17 K to 300 K. The high structural quality of the β-polytype crystals was confirmed through X-ray diffraction, Raman spectroscopy, and high-resolution transmission electron microscopy, demonstrating excellent crystallinity and a nearly stoichiometric In:Se ratio. The temperature-dependent absorption and photoluminescence (PL) spectra are characterized by a prominent free exciton (FX) resonance. At 17 K, the photoluminescence spectrum exhibits a distinct fine-structure splitting of the Wannier–Mott exciton, yielding a triplet state at 1.333 eV and a singlet state at 1.336 eV. Additionally, a biexciton (XX) is localized at an energy of 1.322 eV as confirmed by the nonlinear dependence of intensity on excitation power density. At low temperatures, the absorption spectrum exhibits the free exciton ground state (*n* = 1) at 1.338 eV together with the first excited state (*n* = 2) at 1.350 eV. We systematically tracked and analyzed the temperature evolution of these quasiparticle energies. These findings enhance our understanding of the intrinsic many-body interactions in high-quality InSe, providing essential parameters for advancing its applications in innovative optoelectronic and quantum light-emitting devices.

## 1. Introduction

Layered group-III monochalcogenides, including GaSe, GaS, and InSe, have attracted considerable attention due to their unique electronic and optical properties, which stem from their van der Waals-bonded structures and anisotropic band dispersions. Among these materials, the ε- and β-phases of indium selenide are particularly noteworthy, exhibiting a direct band gap in the near-infrared region (~1.35 eV at low temperature and ~1.25 eV at room temperature) [1,2,3,4,5], high electron mobility [6,7], and enhanced environmental stability compared to other layered semiconductors [8,9]. These characteristics make InSe a promising platform for optoelectronic devices [10,11,12], photovoltaics [13,14,15], and quantum light sources [16,17].

The optical response of InSe is strongly influenced by excitonic effects, owing to its relatively large exciton binding energy (14–20 meV) [18,19]. At low temperatures, photoluminescence (PL) spectra reveal distinct excitonic features, with previous studies reporting peaks attributed to free excitons, donor–acceptor recombinations, and impurity-related transitions [20,21,22,23]. However, despite extensive research on excitonic complexes in two-dimensional transition metal dichalcogenides (TMDs) [24,25,26] and colloidal quantum wells [27,28,29], the finer aspects of exciton physics in bulk InSe including doublet splitting and biexciton formation remain inadequately explored.

Excitonic doublet structures, often referred to as fine-structure splitting (FSS), can arise from electron–hole exchange interactions [30], crystal-field asymmetry [31], or interlayer coupling [32]. Such splitting has been extensively documented in semiconductor quantum dots, where FSS values generally range from a few μeV to several meV. Control over this splitting is crucial for applications in entangled photon emission [33]. In layered GaSe, a related III–VI compound, the singlet–triplet splitting of Wannier–Mott excitons has been reported with energy differences of several meV [34,35]. These findings suggest that excitonic doublet structures may also exist in InSe, although experimental confirmation is limited.

The earliest investigation of the optical properties of InSe single crystals was conducted by Damon and Redington in 1954 [36], wherein the optical absorption was examined in mechanically cleaved layers with thicknesses ranging from 0.5 to 80 μm. In 1968, Andriyashik et al. [37] reported the absorption and reflection of InSe single crystals in the energy region of 1 to 5 eV and in the temperature range of 90 to 293 K. A decade later, Camssel et al. [1] studied the temperature dependence of the excitonic absorption edge from 1.6 to 300 K using a 7-μm-thick InSe sample and observed up to the second excited excitonic state (*n* = 3). In 2003 and 2004, Ates et al. [38,39] investigated the temperature-dependent influence of an external electric field on the absorption edge of InSe and InSe:Ho single crystals, and reported that the application of the field induces a shift of the absorption edge toward lower energies in both samples. Specifically, a shift of about 21 meV at 10 K and 11 meV at room temperature was observed for a 5 kV/cm electric field in the pure InSe sample, while a shift of about 23 meV at 10 K and 17 meV at 240 K was noted for a 7.5 kV/cm electric field in the InSe:Ho sample.

In 1982, Abha and Warrier [21] performed the first photoluminescence (PL) study of InSe single crystals over a temperature range of 12 to 300 K, using an Argon ion laser with a 4880 Å line and 500 mW power output. Three peaks at 1.311, 1.272, and 1.227 eV at 12 K were detected, corresponding to impurity-to-band transitions, donor-acceptor type transitions, and transitions within an impurity vacancy complex. Two years later, Gnatenko et al. [19] examined the luminescence spectrum of InSe single crystals doped with chromium (Cr) at concentration of 0.001% across various temperatures (4.5 to 50 K). Their results showed the free exciton emissions characterized by a doublet structure ($E_{1}^{A}$ = 1.3371 eV and $E_{2}^{A}$ = 1.3365 eV), attributed to valence band splitting due to interlayer interactions. Additionally, two bound exciton states were observed at 1.3343 eV and 1.3234 eV, identified as phonon satellites of the free exciton with optical phonon energies at 17 and 110 cm^−1^, respectively. Recently, Ertap et al. [40] reported PL studies of undoped and boron-doped InSe crystals in the 10–100 K temperature range. At 12 K, PL spectra exhibited four emission bands: 1.337, 1.304, 1.283, and 1.236 eV for pure InSe; 1.335, 1.305, 1.284, and 1.242 eV for 0.1% B-doped InSe; and 1.330, 1.306, 1.284, and 1.244 eV for 0.5% B-doped InSe. These bands are associated with radiative recombination of direct free excitons (*n* = 1), impurity-band transitions, donor–acceptor recombinations, and defect-related transitions involving impurity atoms, vacancy complexes, and structural imperfections.

In this study, we present an in-depth investigation of the optical properties of high-quality β-InSe single crystals from 17 to 300 K. X-ray diffraction, Raman spectroscopy, and high-resolution TEM confirm excellent crystallinity and a near-stoichiometric composition. Temperature-dependent absorption and photoluminescence (PL) spectra exhibit pronounced excitonic signatures, dominated by a strong free-exciton resonance. At 17 K, the PL resolves the Wannier—Mott exciton fine structure into a triplet at 1.333 eV and a singlet at 1.336 eV; a biexciton line appears at 1.322 eV and is verified by its nonlinear power scaling. The absorption spectrum further resolves the free-exciton ground state (*n* = 1, 1.338 eV) and first excited state (*n* = 2, 1.350 eV). Tracking these features across temperature provides new insight into excitonic doublet splitting, biexciton formation, and the thermal stability of quasiparticles in β-InSe.

## 2. Materials and Methods

### 2.1. Sample Growth and Preparation

β-InSe single crystals were grown using a temperature gradient method. A stoichiometric mixture of high-purity indium (In, 99.999%) and selenium (Se, 99.99%) powders in a 1:1 molar ratio was sealed in a cleaned, double-walled quartz ampoule under vacuum (~10^−4^ Torr). The ampoule was placed vertically inside an electric furnace and underwent a multi-stage heating process. It was first heated to 723 K at a rate of 20 K·h^−1^ to initiate the reaction. The temperature was then raised to 1023 K at 10 K·h^−1^ and held for 16 h to complete the reaction. The ampoule was subsequently cooled to 723 K at 1 K·h^−1^, followed by a faster cooling to room temperature at 20 K·h^−1^. The cylindrical part of the grown ingot has a diameter of approximately 15 mm and a length of about 20 mm.

**Figure 1:**
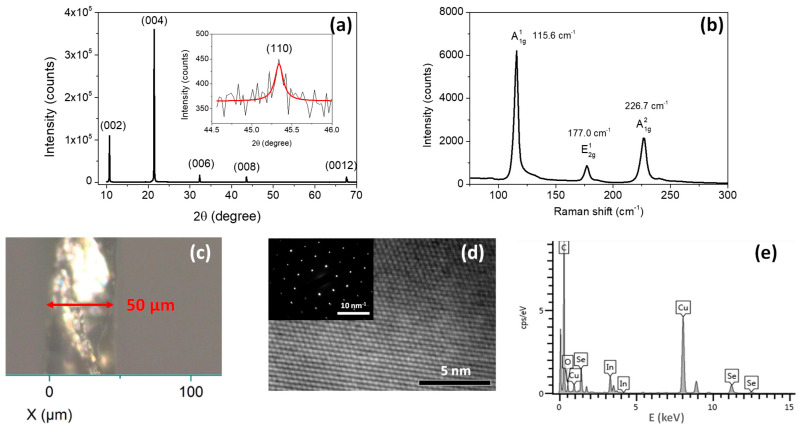
(**a**) XRD pattern of InSe flakes deposited on a glass substrate. (**b**) Raman spectrum of the InSe single crystal characterized on the c-plane. (**c**) Optical image showing the thickness of the InSe thin slab used for the transmission measurements. (**d**) HR-TEM image of the sample captured in the c-plane, with the SAED pattern shown in the inset. (**e**) EDS of the sample.

For absorption measurements, an InSe slab with a thickness of approximately 50 μm was prepared by mechanical exfoliation using Scotch tape, as illustrated in Figure 1c. In addition, InSe flakes were produced via ultrasonic exfoliation in ethanol and subsequently employed for X-ray diffraction (XRD) and transmission electron microscopy (TEM) analyses.

### 2.2. Characterization

X-ray diffraction patterns were recorded on a Bruker D8 Advance diffractometer (Bruker, Billerica, MA, USA) with Cu Kα radiation (λ = 1.5406 Å) over a 2θ range of 10–70° at a scan rate of 2.4°/min. Raman spectra were acquired using an XploRA PLUS spectrometer (Horiba, Kyoto, Japan) with a 532 nm excitation source, a 1% neutral-density filter, and a 2400 grooves/mm grating. The laser was focused with a 100× objective lens (NA = 0.8), yielding a spot size of ~1 μm, and backscattered signals were collected under ambient conditions.

Transmission electron microscopy (TEM) was performed on a JEM-2010 microscope (JEOL, Kyoto, Japan) operating at 200 kV. High-resolution TEM (HRTEM) and selected-area electron diffraction (SAED) were employed to examine the structural and crystallographic properties of the InSe flakes.

Temperature-dependent transmission and photoluminescence (PL) measurements were carried out using a closed-cycle cryostat (APD Cryogenics, Allentown, PA, USA) evacuated to ~10^−6^ mbar (10^−4^ Pa) with a turbomolecular pump. Transmission spectra were obtained with a 50 W halogen lamp through a 2 mm pinhole on the cold finger. PL excitation was provided by a 355 nm laser diode (Teem Photonics, Grenoble, France) at 45° incidence, with an excitation power density of 22.54 W/cm^2^. Emission spectra were collected using an iHR550 spectrometer (Horiba, Kyoto, Japan) equipped with a 150 grooves/mm grating.

## 3. Results and Discussion

The phase purity and structural characteristics of the InSe flakes were first examined by XRD, as shown in Figure 1a. The diffraction peaks at 10.69°, 21.42°, 32.32°, 43.54°, and 67.56° are indexed to the (002), (004), (006), (008), and (0012) planes, characteristic of the hexagonal phase of InSe. These reflections are consistent with earlier reports and correspond well to the standard JCPDS card No. 87-34-1431 [41,42]. In addition, the appearance of the (110) reflection at 45.34° is attributed to the random orientation of exfoliated InSe flakes. The calculated lattice parameters based on the hexagonal crystal system are *a* = 4.00 Å and *c* = 16.62 Å. Raman spectroscopy provided further verification of the crystal structure. As shown in Figure 1b, the Raman spectrum exhibits three characteristic modes: $A_{1g}^{1}$, $E_{2g}^{1}$, and $A_{1g}^{2}$ at 115.6, 177.0 and 226.7 cm^−1^, respectively, which are in excellent agreement with previous studies [43,44]. These vibrational fingerprints unambiguously confirm the β-polytype of InSe. High-resolution TEM analysis in Figure 1d revealed well-resolved lattice fringes of the c-plane, while the corresponding SAED pattern displayed six-fold rotational symmetry, further corroborating the hexagonal lattice. The stoichiometric composition is determined by the energy dispersive X-ray spectrum (EDS), as shown in Figure 1e, with an In:Se ratio of approximately 1.08:1. The C, O, and Cu signals are attributable to the TEM support grid rather than the InSe sample [45].

**Figure 2:**
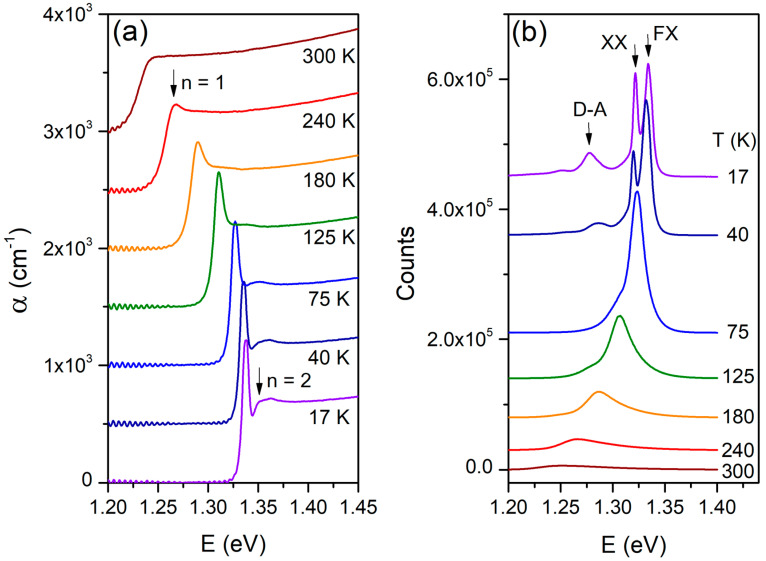
Temperature-dependent (**a**) absorption and (**b**) photoluminescence spectra of the InSe thin slab measured between 17 and 300 K. For clarity, representative spectra at selected temperatures are displayed with vertical offsets.

The temperature-dependent absorption of InSe single crystals in the range of 17 K to 300 K is shown in Figure 2a. The absorption coefficient α was obtained from transmission spectra using the relation $\alpha =1/dlog(I_{0}/I)$, where *d* is the sample thickness as shown in Figure 1c, *I_0_* the transmitted intensity without the sample, and *I* the transmitted intensity with the sample. A clear blue shift and sharpening of the excitonic features and band edge are observed with decreasing temperature. At low temperatures, the excited excitonic state (n = 2) is resolved. The evolution of PL spectra, excited by a 355 nm laser with a power density of 22.54 W.cm^−2^, is presented in Figure 2b. The PL intensity increases markedly as the temperature decreases, with the dominant emission peak assigned to the recombination of free excitons (FX), specifically the n = 1 state associated with the direct band gap, in agreement with previous reports [18,19,21,23,40]. Additional peaks appear at lower energies relative to the FX emission at low temperatures, which may originate from biexcitons (XX), as discussed later. The high-energy FX feature corresponds to the ground states of Wannier-Mott excitons formed by electrons at the conduction band minimum and holes at the valence band maximum at the Γ-point [46,47,48]. A peak at about 1.27 eV is attributed to donor-acceptor pair recombination, while an even lower-energy feature is likely associated with deep-level defect states [21].

**Figure 3:**
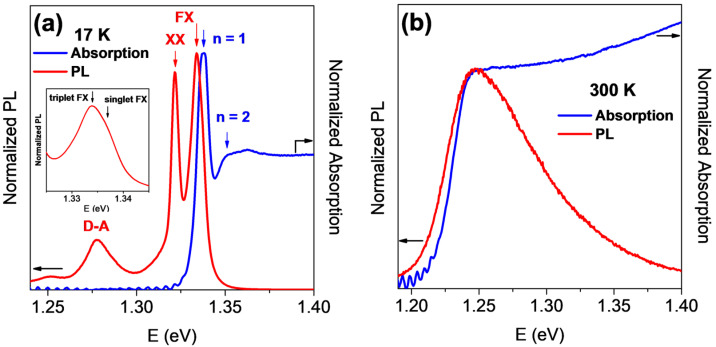
Photoluminescence and absorption spectra of a InSe thin slab obtained at two temperatures: (**a**) 17 and (**b**) 300 K. The inset in (**a**) indicates the singlet and triplet states of PL spectrum at 17 K.

To gain further insight into the low-temperature optical response of InSe single crystals, Figure 3a,b shows the PL and absorption spectra measured at 17 and 300 K, respectively. At 17 K, the free exciton (FX) ground state exhibits a fine-structure splitting into a triplet (1.333 eV) and a singlet (1.336 eV), corresponding to an energy separation of 3 meV. This value is consistent with the earlier report of the γ-InSe given by Paraskevopoulos et al. [49], but significantly larger than the 0.6 meV splitting observed at 4.5 K by Gnatenko et al. [50]. As pointed out by Costa et al. [51], the β- and γ-InSe polytypes exhibit essentially identical electronic properties. Due to the weak Van der Waals nature of the interlayer interactions, the arrangement of the layers does not significantly influence the electronic structure, resulting in similar photoluminescence (PL) properties. The discrepancy may arise from differences in polytype, excitation source, or crystal quality. The origin of this doublet structure has remained unclear until now. In Ref. [50], this feature is considered as a doublet structure due to valence band splitting by interlayer interaction. In contrast, Ref. [49] assignes these features as a bound exciton and an excitonic peak. The clear appearance of the excited state (*n* = 2) is observed in the absorption spectrum at 17 K and a peak in higher energy side is band-to-band absorption. At 300 K, the PL spectrum is broad and asymmetric, featuring an extended high-energy tail accurately modeled by an empirical asymmetric double-sigmoid function [52,53].

The temperature-dependent shifts in the exciton and biexciton energies can be quantitatively described by the following relation:

(1) $$E(T)=E_{0}+AT+B\frac{1}{e^{\hbar \omega_{ph}/k_{B}T}-1},$$

where the linear term represents the contribution from lattice thermal expansion, while the second term accounts for the electron–phonon interaction effects on the electronic energy [54].

**Figure 4:**
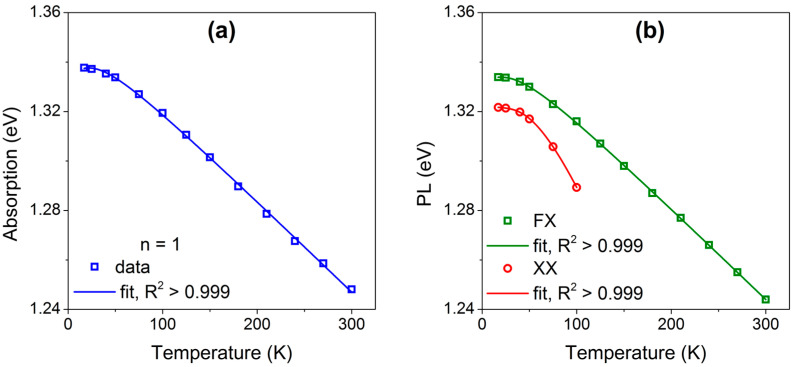
Temperature dependence of the ground-state exciton (squares) and biexciton (circles) energies in InSe single crystal. Experimental data are shown as points; solid lines are fits for (**a**) absorption and (**b**) photoluminescence.

Figure 4a,b shows the temperature dependence of the ground-state exciton and biexciton energies in absorption and PL, respectively; symbols denote experimental data and solid lines are fits. The corresponding best-fit parameters to Equation (1) are listed in Table 1. The fitted phonon energies $\hbar \omega_{ph}$ of the ground exciton states for both absorption and PL is 8.6 ± 3.4 and 10.0 ± 1.2 meV, respectively, comparable to the $A_{1g}^{1}$ optical phonon at 14.3 meV, as shown in Figure 1b. It is worth to emphasize that fitting values of data below 200 K bring the closer results to optical mode $A_{1g}^{1}$ because position of peaks is more identical. The phonon energy associated with the biexciton is in close agreement with the $E_{2g}^{1}$ optical mode (21.5 meV). The redshift of the biexciton occurs more rapidly than that of the exciton, and this phenomenon has also been observed in cubic ZnS single crystals [55] and in the layered structure of Sb_2_Se_3_ [56].

**Figure 5:**
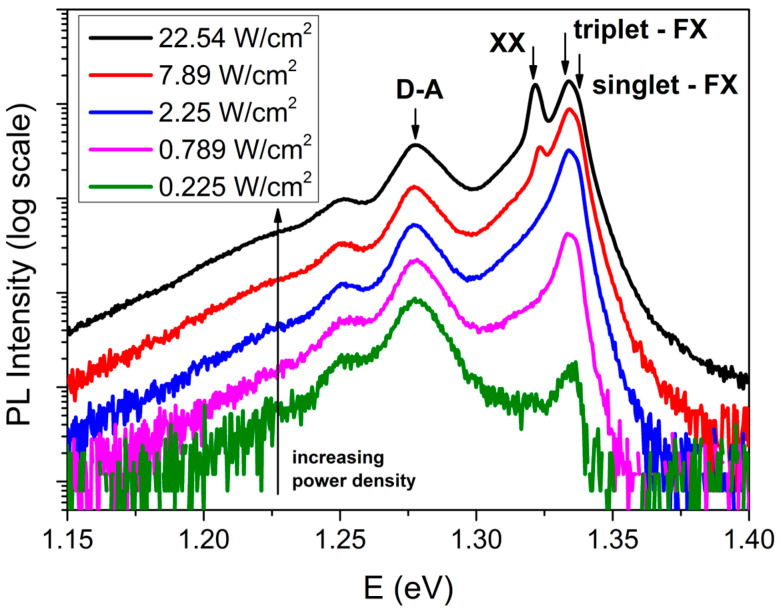
Log-scale luminescence spectra of an InSe single crystal at 17 K, measured under 355 nm excitation as a function of excitation power density.

Figure 5 presents the PL spectra of an InSe single crystal recorded at 17 K under varying excitation power densities. The spectra illustrate the evolution of radiative recombination pathways as the excitation power increases from 0.225 W/cm^2^ to 22.54 W/cm^2^. At lower excitation levels, the emission is primarily dominated by a broad peak centered around 1.275 eV, which can be attributed to donor–acceptor pair (D–A) recombination. As the power density increases, this peak rises rapidly and subsequently saturates, reflecting the characteristic behavior associated with D–A recombination. A similar saturation effect is observed for the feature at approximately 1.25 eV, which is associated with deep-level defect states.

At elevated excitation densities, additional high-energy features emerge, corresponding to excitonic transitions. The peaks designated as “singlet-FX” and “triplet-FX” exhibit an almost linear increase in intensity with excitation power, thereby confirming their identification as free exciton recombination. At sufficiently high power densities, a new peak appears at 1.322 eV (labeled “XX”), attributed to biexciton emission. The formation of biexcitons necessitates a substantial exciton population, making this feature prominent only under strong excitation conditions. The detection of biexciton emission at 17 K aligns with previous findings reported at 5 K [46].

## 4. Conclusions

In conclusion, we investigated the optical properties of high-quality β-InSe single crystals over a temperature range of 17–300 K. Structural analyses using XRD, Raman spectroscopy, and HRTEM confirmed excellent crystallinity and near-stoichiometric composition. Temperature-dependent absorption and photoluminescence spectra revealed strong excitonic features dominated by free exciton resonances. At 17 K, the PL spectra clearly showed a fine-structure splitting of the Wannier–Mott exciton into a triplet state at 1.333 eV and a singlet state at 1.336 eV, corresponding to a separation of approximately 3 meV. Additionally, a biexciton emission at 1.322 eV was identified and verified through its nonlinear dependence on excitation power density. The temperature evolution of the exciton and biexciton peaks exhibited a redshift behavior consistent with lattice expansion and electron–phonon interactions. These findings provide direct evidence of excitonic doublet splitting and biexciton formation in bulk β-InSe, underscoring its potential for future optoelectronic and quantum photonic applications.

## Figures and Tables

**Table 1:** Parameters from the best fits to Equation (1) describing the temperature-dependent exciton and biexciton energies in absorption and PL.

	Exciton States	$E_{0}$ (eV)	A (meVK)	B (meVK)	$\hbar \omega_{ph}$ (meV)
Absorption	n=1	1.336 ± 0.005	0.1 ± 0.2	−47 ± 7	8.6 ± 3.4
PL	FX (n=1)	1.333 ± 0.001	0.04 ± 0.06	−47 ± 2	10.0 ± 1.2
	XX	1.322 ± 0.001	−0.03 ± 0.01	−194 ± 13	17.4 ± 0.7

## Data Availability

The raw data supporting the conclusions of this article will be made available by the authors on request.
